# Participatory assessment of management and biosecurity practices of smallholder pig farms in North East India

**DOI:** 10.3389/fvets.2023.1196955

**Published:** 2023-07-03

**Authors:** Mahak Singh, Nungshitula Pongenere, R. T. Mollier, R. N. Patton, Rekha Yadav, Rahul Katiyar, Priyanka Jaiswal, M. Bhattacharjee, H. Kalita, V. K. Mishra

**Affiliations:** ^1^Animal Reproduction Laboratory, ICAR Research Complex for NEH Region, Nagaland Centre, Medziphema, Nagaland, India; ^2^Department of Agronomy, School of Agricultural Sciences and Rural Development, Nagaland University, Medziphema Campus, Lumami, India; ^3^Division of Animal Health and Fisheries Science, ICAR Research Complex for NEH Region, Umiam, Meghalaya, India; ^4^Amity School of Economics, Amity University, Sector-125, Noida, Uttar Pradesh, India; ^5^ICAR Research Complex for NEH Region, Nagaland Centre, Medziphema, Nagaland, India; ^6^ICAR Research Complex for NEH Region, Umiam, Meghalaya, India

**Keywords:** smallholder pig farms, health, risk factors, biosecurity, India

## Abstract

The present study was aimed at describing the pig production system, farm management, pig movement, and existing biosecurity level of smallholders' pig production system in North East India. A cross-sectional survey of 1,000 pig producers in four districts (two urban and two rural) in core pig-producing regions of India, where ASF occurrence had been reported, was conducted. The mean pig population was significantly (*p* < 0.05) higher in urban districts. In urban districts, most of the pig houses were isolated but located on the roadside, while in rural districts, commune pig houses along the roadside were more common. The majority of the respondent purchased (91%) or sold (60%) the pigs during the past 12 months. Swill feeding was common in the entire study area. The majority of the respondent (80%) in rural districts were unaware of ASF. Significant pig trade of live pigs and pork products was observed in the urban district. In the case of on-farm biosecurity measures, only 6.9% of respondents had fencing for the pig farm, 99.3% did not have provision for a footbath, and only 17.2% of the respondents restricted visitors' access to the pig farm. The study revealed that the pig production system is dominated by smallholding units with a frequent introduction or exit of pigs along with poor on-farm biosecurity measures. With the current level of farm management and biosecurity practices, smallholder pig farmers are at an increased risk of ASF and other contagious diseases.

## 1. Introduction

In India, pigs are reared by vulnerable or disadvantaged communities for income generation and food and nutritional security (1–3). Pork is a cheap source of protein for them. Pig-rearing is considered a means of poverty alleviation in these low-income settings (4). The North East Hill (NEH) region of India has 46% of India's total pig population, mostly reared by smallholder pig farmers. The high density of the pig population in the NEH region of India with low biosecurity measures provides favorable opportunities for the spread of Afric surveillance and reporting system is weak, leading to under-reporting of disease outbreaks. The NEH region of India shares a long and porous international boundary with China, Myanmar, Nepal, Bhutan, and Bangladesh. Also, the trade of live pigs, pork, feed, etc. from other states (5), as well as neighboring countries, is poorly regulated in this region, thereby further increasing the risk of disease spread.

African swine fever (ASF) is a highly contagious viral hemorrhagic transboundary disease with high mortality in domestic pigs and wild boars (6, 7). ASF is caused by the African swine fever virus belonging to the genus *Asfivirus* within the *Asfaviridae* family (8–10). ASF can cause 100% mortality within a few days when introduced into a non-infected pig farm (7). Although the disease is non-zoonotic and has a limited host range, the ASF virus persists for a long duration in live or dead tissues or fomites, and because of this, the disease has spread extensively and affected the pig industry globally (6, 10). In August 2018, ASF was first reported in China and thereafter it has spread to more than 10 countries in Asia including India where the disease has severely affected the smallholder pig farmers in the region (9, 11). In India, the disease was reported in January 2020 in Arunachal Pradesh, a North Eastern hilly (NEH) state of India that shares an international boundary with China (11). The route and origin of ASF in India is still not clear but it was suggested that the disease might have come from China through the wild boar (12). Now, the disease has spread to other NEH states of India and has caused huge economic losses to the smallholder pig producers.

Smallholder pig producers, who are less likely to implement stringent biosecurity measures, are at increased risk of ASF (13). It was previously reported that areas with a high level of pig-related activities tend to have a higher prevalence of ASF (14, 15). In the absence of effective treatments and vaccines, implementing stringent biosecurity systems at the farm and community levels are the only effective strategies to contain the disease (6, 13). There is limited research on pig production systems, management practices, pig health management, and biosecurity measures in North East India. Considering the importance and vulnerability of pigs in core pig-producing regions, insight into the strengths and weaknesses of smallholders' pig farms is therefore essential. Therefore, the present study was aimed at describing the pig production system, management practices, and existing biosecurity level in North East India.

## 2. Materials and methods

### 2.1. Site selection and sample size

**Figure 1:**
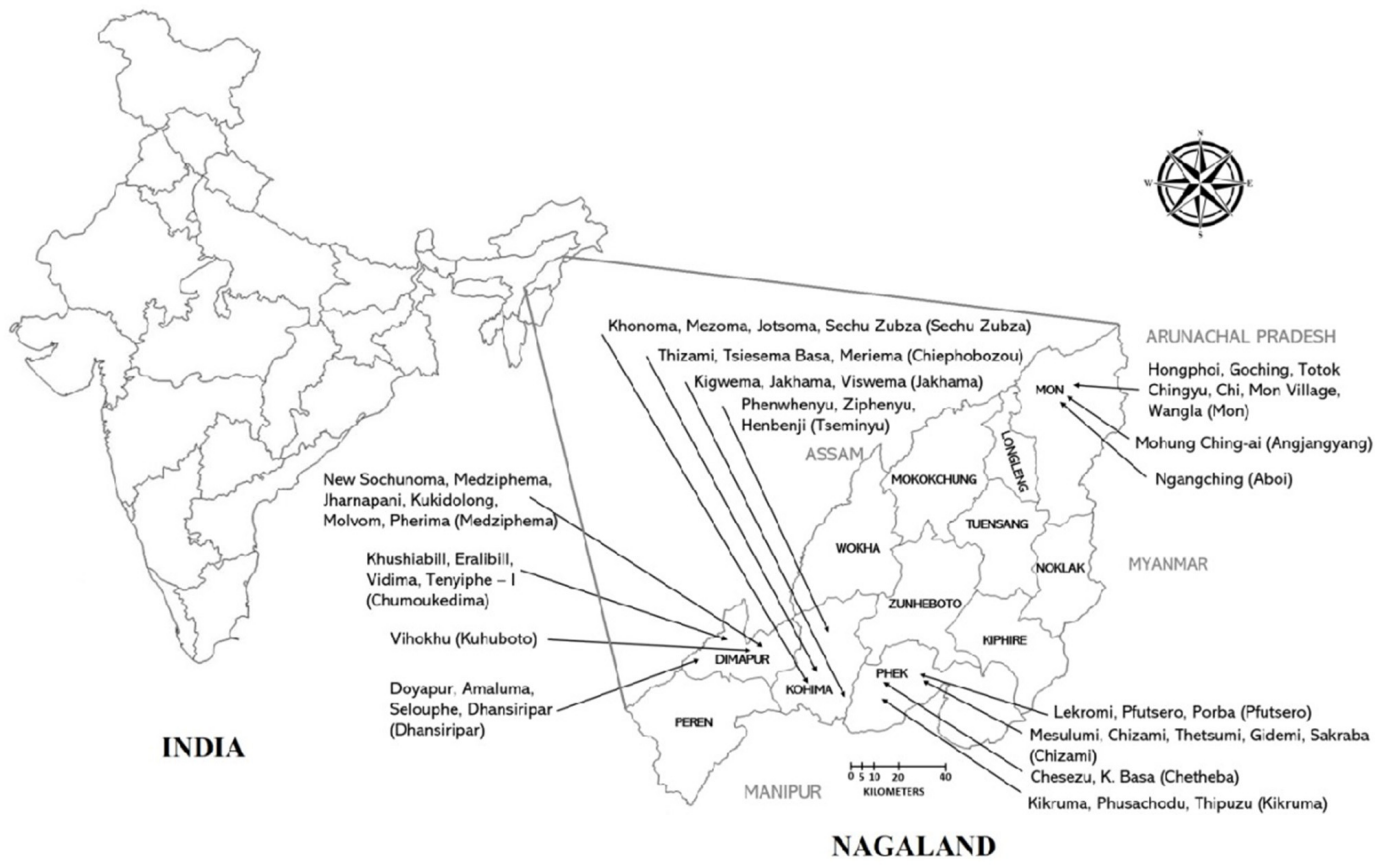
The map of India (with states) and Nagaland (with districts) showing study locations in detail. Villages are mentioned with arrow in each district. The name of the block is written in parentheses.

The study was conducted between January 2021 to May 2022 in Nagaland, a North Eastern Hilly (NEH) state of India (Figure 1). The study site was selected purposefully as pig density, per capita pork consumption, and pig trades are highest in Nagaland (5). Also, ASF outbreaks have been reported in the study area. Similarly, Ma et al. (16) reported that pig density is the most important predictor of ASF outbreaks. Four districts were selected for the present study, two rural districts (Phek and Mon) and two urban districts (Dimapur and Kohima). Dimapur and Kohima are the largest urban centers with the highest pig population as well as significant trade of live pig and pork. Phek and Mon districts are rural areas and share an international boundary with Myanmar. The selected districts are high-risk locations for ASF outbreaks because of the high trade of live pig and pork, high consumption of pork, porous international border, and close proximity to the forest. Further selections of blocks were done based on the latest National Livestock Census data (maximum pig population). The villages were selected randomly from selected blocks by the research team. The list of selected blocks and villages in each district is given in Table 1 and Figure 1. Households were selected randomly in each village in consultation with the village council. For this, the meeting of the village council was called by the village chairman wherein the study team also participated and briefed the village council about the scope of the study. Thereafter, the village council prepared a list of households that were rearing pigs. The households for the study were randomly selected from the list by the research team and no two adjacent households were selected. All the respondents were informed about the study and their oral consent was taken. Proper biosecurity and sanitary measures were taken to avoid the spread of infection between the farms. These include the use of disinfectants (Sodium hypochlorite), disposable gumboot covers, aprons, and hand sanitizers. The households having sick pigs at the time of the visit were not interviewed to avoid the spread of infection. As such, a total of 1,000 households that were rearing pigs were surveyed in all four districts. The sample size was decided as per Thrusfield (17). In short, 70% of the population in the research region raised pigs, and the projected sample size for each district was 227 assuming a 90% confidence range and a 5% level of accuracy.

**Table 1:** Details of the selected districts, blocks, and villages for survey work.

**Districts**	**Blocks**	**Villages**	**Households (nos.)**	**Number of pigs**
Dimapur	Chumoukedima, Medziphema, Dhansiripar, Kuhoboto	Amaluma, Dhansiripar, Doyapur, Eralibill, Jharnapani, Kushiabill, Kukidolong, Molvom, Medziphema, New Sochunoma, Pherima, Selouphe, Tenyiphe-(I), Vihokhu, Vidima,	250	1,060
Mon	Mon, Aboi, Angjangyang	Chi, Goching, Hongphoi, Mohung Ching-ai, Mon vill, Ngangching, Totokchingyu, Wangla	250	569
Phek	Pfutsero, Kikruma, Chizami, Chetheba	Chesezu, Chizami, Gidemi, K Basa, Kikruma, Lekromi, Mesulumi, Pfutsero, Phusachadu, Porba, Sakraba, Thetsumi, Thipuzu	250	884
Kohima	Tseminyu, Jakhama, Sechu-Zubza, Chiephobozou	Henbenji, Jakhama, Jotsoma, Khonoma, Kigwema, Meriema, Mezoma, Phenwhenyu, Thizama, Thizami, Tsiesema, Viswema, Ziphenyu, Zubza	250	1,042
Total	15	50	1,000	3,555

### 2.2. Questionnaire design and data collection

A semi-structured questionnaire in English was developed by the authors based on field observations, interviews with pig farmers, discussions with veterinary officers, and relevant literature (7, 15). The questionnaire included information on the demography of farmers, pig husbandry and management, biosecurity measures, diseases and mortality, and the movement of live or dead pigs. The survey was piloted in two villages in the Dimapur district using 10 households (five from each village) by two local interviewers in local dialects (Nagamese). Piloted survey households were not included in the final study. Following the pilot, the questionnaire was modified to improve clarity. For the final study, the questionnaire was administered in local dialects to the respondent by the study team (Konyak in Mon; Tenyidie in Phek and Kohima; Nagamese in Dimapur). In 1 day, data were collected from one village only and ~30–45 min were spent to collect data from one household. Data on diseases outbreak and mortality were triangulated with a local veterinary field assistant and veterinarian. To encourage the participation of farmers and build rapport with them, participants were provided with 500 g of mineral mixture for pigs at the start of the interview. The information collected was daily entered in Microsoft Excel and cross-examined by the lead author for any errors.

### 2.3. Data analysis

Statistical analysis was performed using Stata 14.2 (Stata Corporation, Texas USA) and Microsoft Excel (Microsoft Corporation, USA) software.

## 3. Results

### 3.1. Demographic characteristics of the respondents

The study was conducted in four districts, covering 15 blocks and 50 villages. In total, 1,000 respondents were interviewed for the study. The average age of respondents was 50.51 years (Table 2) and most of them were men (79.5%). The majority of the participants were within the 31–60 years age group (78.9%). In total, approximately 80% of the respondents had a level of education up to primary school. The rural district (Mon) had the highest number of illiterate respondents (19.3%). The primary activity for most respondents interviewed was mixed farming involving crop, horticulture, livestock, and poultry (59%). In rural districts (Mon and Phek), around two-thirds of the farmers were engaged in mixed farming. Pig rearing as the primary activity was reported by only 7.6% of respondents. The majority of the respondents (81.8%) in rural districts had *kutcha* (made up of bamboo, wood with earthen floor) houses for dwelling while in urban districts, 28.2% of the respondents had concrete houses. The majority of the respondents also had poultry (95.4%) while cattle, goat, and mithun (*Bos frontalis*) were also kept by a few respondents. Approximately 81% of respondents did not attend any training program on pig health management. The mean pig population was significantly (*p* < 0.05) higher in urban districts (4.17–4.24 pigs) compared to rural (2.28–3.54 pigs) districts (Table 3). In rural districts, respondents had maximum numbers of castrated boar (1.29–1.36) and grower pigs (0.58–1.11) compared to other categories of pigs.

**Table 2:** Demographic characteristics of pig keepers in the selected districts.

**Variable**	**Dimapur *n =* 250**	**Kohima *n =* 250**	**Phek *n =* 250**	**Mon *n =* 250**	**Total *n =* 1,000**
Mean age in years (mean ± SD)	52.21 ± 9.76	53.14 ± 10.64	49.62 ± 12.64	47.08 ± 12.11	50.51 ± 11.57
**Age group**					
≤ 30 years	0 (0)	0	12 (4.8)	11(4.4)	23 (2.3)
31–60 years	200 (80)	200 (80)	190 (76)	199 (79.6)	789 (78.9)
>61 years	50 (20)	50 (20)	48 (19.2)	40 (16)	188 (18.8)
Total	250 (100)	250 (100)	250 (100)	250 (100)	1,000 (100)
**Sex**					
Male	180 (72)	205 (82)	198 (79.2)	212 (84.8)	795 (79.5)
Female	70 (28)	45 (18)	52 (20.8)	38 (15.2)	205 (20.5)
**Education**					
Primary	55 (22)	72 (28.8)	83 (33.2)	68 (27.2)	278 (27.8)
Secondary	152 (60.8)	122 (48.8)	108 (43.2)	91 (36.4)	473 (47.3)
Graduation	21 (8.4)	23 (9.2)	12 (4.8)	0 (0)	56 (5.6)
Illiterate	22 (8.8)	33 (13.2)	47 (18.8)	91 (36.4)	193 (19.3)
Total	250 (100)	250 (100)	250 (100)	250 (100)	1,000 (100)
**Primary activity**					
Pig rearing	26 (10.4)	28 (11.2)	8 (3.2)	14 (5.6)	76 (7.6)
Crop farming	22 (8.8)	30 (12)	40 (16)	37 (14.8)	129 (12.9)
Mixed farming	93 (37.2)	127 (50.8)	181 (72.4)	189 (75.6)	590 (59)
Others	109 (43.6)	65 (26)	21 (8.4)	10 (4)	205 (20.5)
Total	250 (100)	250 (100)	250 (100)	250 (100)	1,000 (100)
**Farmers' housing type**					
Kutcha (bamboo, wood, thatched roof)	51 (20.4)	95 (38)	187 (74.8)	222 (88.8)	555 (55.5)
Pucca (concrete)	88 (35.2)	53 (21.2)	37 (14.8)	17 (6.8)	195 (19.5)
Mixed	111 (44.4)	102 (40.8)	26 (10.4)	11 (4.4)	250 (25)
Total	250 (100)	250 (100)	250 (100)	250 (100)	1,000 (100)
**Farmers who kept other animals**					
Cattle	65 (26)	42 (16.8)	7 (2.8)	4 (1.6)	118 (11.8)
Poultry	245 (98)	235 (94)	242 (96.8)	232 (92.8)	954 (95.4)
Goat	15 (6)	5 (2)	4 (1.6)	2 (0.8)	26 (2.6)
Mithun	0 (0)	7 (2.8)	12 (4.8)	2 (0.8)	21 (2.1)
**Training on pig health management**					
Yes	72 (28.8)	78 (31.2)	22 (8.8)	13 (5.2)	185 (18.5)
No	178 (71.2)	172 (68.8)	228 (91.2)	237 (94.8)	815 (81.5)
Total	250 (100)	250 (100)	250 (100)	250 (100)	1,000 (100)

**Table 3:** Pig population structure in the selected districts (Mean ± SD).

	**Urban**		**Rural**	
	**Dimapur**	**Kohima**	**Phek**	**Mon**
Sows	0.54 ± 0.61	0.32 ± 0.65	0.12 ± 0.44	0.06 ± 0.27
Boars	0.08 ± 0.28	0.05 ± 0.22	0.02 ± 0.15	0.04 ± 0.20
Castrated boars	1.66 ± 0.94	1.06 ± 0.74	1.29 ± 0.70	1.36 ± 0.79
Growers	1.03 ± 0.79	1.47 ± 1.30	1.11 ± 0.99	0.58 ± 0.65
Piglets	0.92 ± 1.34	1.27 ± 0.88	0.99 ± 1.32	0.23 ± 0.96
Mean Pig population	4.24 ± 1.28^a^	4.17 ± 1.98^a^	3.54 ± 1.57^b^	2.28 ± 1.63^c^

Mean bearing different superscipt in a row differ significantly (*p* < 0.05).

### 3.2. Pig husbandry practices

The majority of the respondents (83%) kept pigs for fattening purposes and only 1.9% of the farmers reared pigs for breeding purposes (Table 4). Rearing pigs for breeding was least preferred in rural districts. The majority of respondents in the urban districts kept crossbred pigs (80% in Dimapur and 70.4% in Kohima), whereas, in the rural districts, the majority of the respondent kept a local breed of pigs (54.8% in Phek and 52.9% in Mon). Pig pens made of wood were more prevalent in the rural districts (63.6% in Phek and 68.8% in Mon), while concrete pig pens were more common in the urban districts (67.2% in Dimapur and 70.4% in Kohima). In the urban districts, most of the pig houses were isolated but located on the roadside (individual pig houses located on the main road) (54% in Dimapur and 61.2% in Kohima), while in rural districts, commune pig houses (the common pig housing system adopted by a group of farmers) along the roadside were more common (44.4% in Phek and 26.8% in Mon). The majority of the respondents purchased (91%) or sold (60%) pigs during the past 12 months across the study region. In the urban districts, the majority of the respondents (48.8% in Dimapur and 54.8% in Kohima) reported trading activities three to five times, while in the rural districts, it was one to two times (56.4% in Phek and 64.8% in Mon) in the past 1 year. Swill feeding was practiced by the majority of the respondents (78.5%) in urban as well as rural districts. In total, approximately 67% of the respondents were involved in the hunting of wild boar in the past 1 year. The majority of the respondents (65%) in the urban districts had heard of ASF, while in the rural districts, approximately 80% of the respondents were not aware of ASF.

**Table 4:** Pig husbandry practices practiced by smallholder pig farms in India.

		**Urban**		**Rural**		
**Variable**	**Category**	**Dimapur (%)** ***n** =* **250**	**Kohima(%)** ***n** =* **250**	**Phek(%)** ***n** =* **250**	**Mon(%)** ***n** =* **250**	**Total** ***n** =* **1,000**
Reason for rearing pig	Fattening	180 (72)	193 (77.2)	229 (91.6)	237 (94.8)	839 (83.9)
	Breeding	12 (4.8)	4 (1.6)	1 (0.4)	2 (0.8)	19 (1.9)
	Breeding and fattening	58 (23.2)	53 (21.2)	20 (8)	11 (4.4)	142 (14.2)
	Total	250 (100)	250 (100)	250 (100)	250 (100)	1,000 (100)
Pig breed	Local	37 (14.8)	53 (21.2)	137 (54.8)	148 (59.2)	375 (37.5)
	Exotic	13 (5.2)	21 (8.4)	7 (2.8)	4 (1.6)	45 (4.5)
	Crossbred	200 (80)	176 (70.4)	106 (42.4)	98 (39.2)	580 (58)
	Total	250 (100)	250 (100)	250 (100)	250 (100)	1,000 (100)
Pigpen type	Kutcha (earthen flooring)	0 (0)	4 (1.6)	7 (2.8)	12 (4.8)	23 (2.3)
	Pucca (concrete)	168 (67.2)	176 (70.4)	84 (33.6)	66 (26.4)	494 (49.4)
	Kutcha (wooden)	82 (32.8)	70 (28)	159 (63.6)	172 (68.8)	483 (48.3)
	Total	250 (100)	250 (100)	250 (100)	250 (100)	1,000 (100)
Location of the pig pen	Isolated and away from the roadside	86 (34.4)	67 (26.8)	40 (16)	52 (20.8)	245 (24.5)
	Isolated and near the roadside	135 (54)	153 (61.2)	112 (44.8)	87 (34.8)	487 (48.7)
	Grouped with other farmers	29 (11.6)	30 (12)	98 (39.2)	111 (44.4)	268 (26.8)
	Total	250 (100)	250 (100)	250 (100)	250 (100)	1,000 (100)
Pig trade (purchased/sold) in the last 12 months	Purchased	232 (92.8)	218 (87.2)	238 (95.2)	231 (92.4)	919 (91.9)
	Sold	122 (48.8)	156 (62.4)	167 (66.8)	102 (40.8)	602 (60.2)
In the past 1 year, how many times have pig/piglets been purchased or sold	1-2 times	81 (32.4)	67 (26.8)	141 (56.4)	162 (64.8)	451 (45.1)
	3-5 times	122 (48.8)	137 (54.8)	87 (34.8)	77 (30.8)	423 (42.3)
	More than 5 times	47 (18.8)	46 (18.4)	22 (8.8)	11 (4.4)	126 (12.6)
	Total	250 (100)	250 (100)	250 (100)	250 (100)	1,000 (100)
Source of water for pig	Tap	103 (41.2)	98 (39.2)	42 (16.8)	34 (13.6)	277 (27.7)
	Well	112 (44.8)	114 (45.6)	172 (68.8)	181 (72.4)	579 (57.9)
	Spring, rainwater, river	35 (14)	38 (15.2)	36 (14.4)	35 (14)	144 (14.4)
	Total	250 (100)	250 (100)	250 (100)	250 (100)	1,000 (100)
Feeding practices	Swill feeding	97 (38.8)	79 (31.6)	126 (50.4)	122 (48.8)	424 (42.4)
	Swill feeding+rice+wheat bran	57 (22.8)	62 (24.8)	28 (11.2)	20 (8)	167 (16.7)
	Swill feeding+ wild fodder	19 (7.6)	15 (6)	72 (28.8)	88 (35.2)	194 (19.4)
	Commercial feed	77 (30.8)	94 (37.6)	24 (9.6)	20 (8)	215 (21.5)
	Total	250 (100)	250 (100)	250 (100)	250 (100)	250 (100)
In the past 1 year, have you hunted wild boar	Yes	68 (27.2)	122 (48.8)	202 (80.8)	235 (94.0)	627 (62.7)
	No	182 (72.8)	128 (51.2)	48 (19.2)	15 (6.0)	373 (37.3)
	Total	250 (100)	250 (100)	250 (100)	250 (100)	1,000 (100)
Have you heard of ASF?	Yes	171 (68.4)	155 (62)	54 (21.6)	36 (14.4)	416 (41.6)
	No	79 (31.6)	95 (38)	196 (78.4)	214 (85.6)	584 (58.4)
	Total	250 (100)	250 (100)	250 (100)	250 (100)	1,000 (100)

### 3.3. Diseases occurrence in pigs during the last 1 year

**Figure 2:**
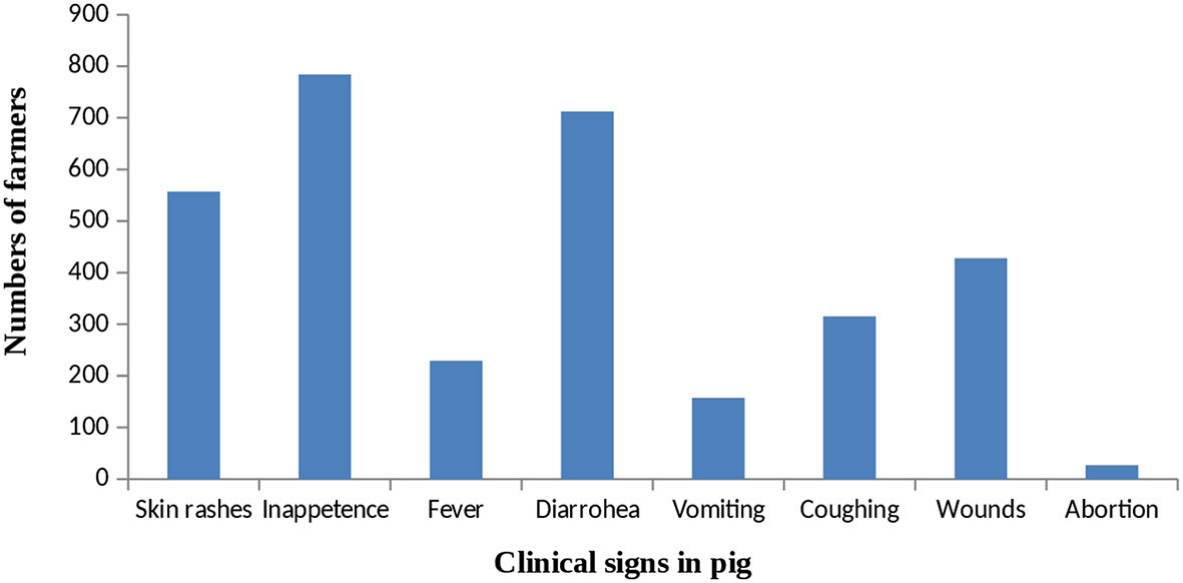
Frequency of clinical signs in pigs as reported by respondents.

The majority of the respondents reported that inappetence (78.4%), diarrhea (71.2%), and skin rashes (55.7%) are common in their pigs (Figure 2). The occurrence of wound, fever, and coughing was reported by 42, 22, and 31% respondents, respectively. In the past 1 year, 231 farmers reported the death of adult pigs (more than 1 year of age), 472 farmers reported the death of grower pigs (2–12 months of age), and 182 farmers reported the death of piglets (<2 months of age). Abortions were reported by only 2.7% of respondents.

### 3.4. Basic biosecurity measures on pig farms

Respondents were administered 19 questions on basic biosecurity measures to be adopted on pig farms. In all four districts, basic biosecurity measures were poorly implemented on pig farms. Only 6.9% of respondents fenced their pig farm with the provision of a gate (Table 5). The majority of the respondents (99.3%) did not have the provision of a footbath at the entrance of the pig farm. Restricted access of visitors to the farm was reported by 17.2% of the respondents; however, in the rural districts, it was only 4.8 and 8.4%. Only 2% of respondents practiced quarantine of sick animals, while 1.1% of respondents quarantined newly purchased pigs. The regular cleaning of the pig pen was reported by 64% of respondents; however, only 10% of respondents used disinfectant. The majority of the respondents (72.6%) from the urban districts reported regular cleaning of feeders and drinkers, while in the rural districts, the majority of the respondents (70.6%) did not clean the feeders and drinkers. Only 15% of the respondents reported the safe disposal of manure and dead pigs. Most of the respondents (80%) purchased pigs or piglets from an unknown source. More than 90% of the respondents did not change their clothes before and after working on the pig farm and did not use separate slippers or gum boots. Only 1.9% of respondents had an all-in all-out production system, while in the rural districts, none of the respondents practiced an all-in all-out production system. Most of the respondents (93%) reported no waste after slaughter of the pigs. Only 21.7% of respondents informed a Veterinarian or Veterinary Field Assistant during a disease outbreak or death in pigs. Record-keeping was practiced by 3.6% of the respondents only. More than 90% of the respondents kept different categories of pigs together and did not have a facility for the isolation of different animal species. Only 4% of the respondents reported the adoption of rodent control measures on the farm.

**Table 5:** Response to basic biosecurity measures in pig farm of respondents.

			**Urban**		**Rural**		
**SI No**.	**Biosecurity measures**	**Category**	**Dimapur (%)**	**Kohima (%)**	**Phek (%)**	**Mon (%)**	**Total (%)**
1.	Fencing of the farm with the provision of a gate	Yes	32 (12.8)	20 (8)	8 (3.2)	9 (3.6)	69 (6.9)
		No	218 (87.2)	230 (92)	242 (96.8)	241 (96.4)	931 (93.1)
2.	Footbath	Yes	5 (2)	2 (0.8)	0 (0)	0 (0)	7 (0.7)
		No	245 (98)	248 (99.2)	250 (100)	250 (100)	993 (99.3)
3.	Restricted access to visitors	Yes	67 (26.8)	72 (28.8)	12 (4.8)	21 (8.4)	172 (17.2)
		No	183 (73.2)	178 (71.2)	238 (95.2)	229 (91.6)	828 (82.8)
4.	Quarantine of sick animals	Yes	8 (3.2)	10 (4)	2 (0.8)	0 (0)	20 (2)
		No	242 (96.8)	240 (96)	248 (99.2)	250 (100)	980 (98)
5.	Quarantine of newly purchased animals	Yes	4 (1.6)	7 (2.8)	0 (0)	0 (0)	11 (1.1)
		No	246 (98.4)	243 (97.2)	250 (100)	250 (100)	989 (98.9)
6.	Regular cleaning (daily)	Yes	212 (84.8)	198 (79.2)	112 (44.8)	117 (46.8)	639 (63.9)
		No	38 (15.2)	52 (20.8)	138 (55.2)	133 (53.2)	361 (36.1)
7.	Use of disinfectant on the farm	Ye	46 (18.4)	28 (11.2)	18 (7.2)	9 (3.6)	101 (10.1)
		No	204 (81.6)	222 (88.8)	232 (92.8)	241 (96.4)	899 (89.9)
8.	Regular cleaning of feeders and drinkers	Yes	198 (79.2)	165 (66)	80 (32)	67 (26.8)	510 (51)
		No	52 (20.8)	85 (34)	170 (68)	183 (73.2)	490 (49)
9.	Safe disposal of dead pigs and manure	Yes	48 (19.2)	64 (25.6)	18 (7.2)	22 (8.8)	152 (15.2)
		No	202 (80.8)	186 (74.4)	232 (92.8)	228 (91.2)	848 (84.8)
10.	Purchase of pigs or piglets from a known source	Yes	48 (19.2)	65 (26)	32 (12.8)	48 (19.2)	193 (19.3)
		No	202 (80.8)	185 (74)	218 (87.2)	202 (80.8)	807 (80.7)
11.	Change of clothing before and after working on the pig farm	Yes	12 (4.8)	27 (10.8)	8 (3.2)	5 (2)	53 (5.3)
		No	238 (95.2)	223 (89.2)	242 (96.8)	245 (98)	947 (94.7)
12.	Use of separate slippers or gum boots for the pig farm	Yes	22 (8.8)	44 (17.6)	12 (4.8)	19 (7.6)	97 (9.7)
		No	228 (91.2)	206 (82.4)	238 (95.2)	231 (92.4)	903 (90.3)
13.	All-in all-out production system	Yes	8 (3.2)	11 (4.4)	0 (0)	0 (0)	19 (1.9)
		No	242 (96.8)	239 (95.6)	250 (100)	250 (100)	981 (98.1)
14.	Disposal of waste after the slaughter of a pig	No waste	212 (84.8)	225 (90)	245 (98)	248 (99.2)	930 (93)
		Disposed in open	32 (12.8)	12 (4.8)	5 (2)	2 (0.8)	51 (5.1)
		Disposed in drainage	0 (0)	12 (4.8)	0 (0)	0 (0)	12 (1.2)
		Buried	6 (2.4)	1 (0.4)	0 (0)	0 (0)	7 (0.7)
15.	Inform veterinarian or veterinary field assistant during disease or death in pig	Yes	65 (26)	83 (33.2)	42 (16.8)	27 (10.8)	217 (21.7)
		No	185 (74)	167 (66.8)	208 (83.2)	223 (89.2)	783 (78.3)
16.	Record keeping	Yes	12 (4.8)	17 (6.8)	4 (1.6)	3 (1.2)	36 (3.6)
		No	238 (95.2)	233 (93.2)	246 (98.4)	247 (98.8)	964 (96.4)
17.	Do not mix different ages of pigs	Yes	37 (14.8)	28 (11.2)	0 (0)	0 (0)	65 (6.5)
		No	213 (85.2)	222 (88.8)	250 (100)	250 (100)	935 (93.5)
18.	Do not mix different animal species	Yes	65 (26)	35 (14)	0 (0)	0 (0)	100 (10)
		No	185 (74)	215 (86)	250 (100)	250 (100)	900 (90)
19	Rodent control	Yes	18 (7.2)	22 (8.8)	0 (0)	0 (0)	40 (4)
		No	232 (92.8)	228 (91.2)	250 (100)	250 (100)	960 (96)

## 4. Discussion

In India, pigs are reared by socially and economically disadvantaged communities. Pigs are an important source of food and nutritional security to these communities as it provides them with a cheap source of quality animal protein (3, 18). However, recent outbreaks of ASF in core pig production areas in India pose serious threats to the economic, food, and nutritional security of poor households. If timely precautionary measures are not taken, ASF may cause huge economic losses and adverse social impacts on resource-poor pig farmers (9). In view of recent ASF outbreaks in the study area, this study provided baseline information on pig production systems, pig trade, gaps in on-farm biosecurity measures, and risk factors for ASF outbreaks.

In the traditional pig production system, understanding of socioeconomic and cultural practices of smallholder pig farmers is important to devise an effective ASF control strategy (6, 19, 20). In the present study, the vast majority of the respondents were men and between 31–60 years of age. Although economic indicators were not assessed in this study, most of the respondents had *kutcha* houses and field observation indicated that they belonged to the poor section of the society. It was earlier reported that educated farmers are well aware of the scientific management of pigs including health management (7, 20). Leslie et al. (20) reported that the development of communication between animal health workers and farmers is important to improve farmers' knowledge and animal health in the smallholder sector. Most of the respondents practiced mixed farming as their primary activity and they did not take any formal training on piggery. In resource-poor regions, practicing mixed farming including livestock and poultry is a low-investment enterprise for poor farmers and this also diversifies their risk (5, 18, 20).

The mean pig population was 2.28 and 4.24 pigs in the rural and urban districts, respectively. The farmers in developing countries have less economic capacity to keep more numbers of pigs and pig raising is dominated by smallholder pig herds (1, 2, 15, 20). The urban districts are well connected to other parts of the country and are a significant trading hub for live pigs and piglets (5). In the present study, respondents in urban areas had more sows and boars. The respondents from the urban districts had access to government institutes working on pig production located in the region and thereby had knowledge of the rearing of pigs for breeding purposes (2). However, the movement of boar or sow for breeding purposes may aid in the spread of infectious diseases in a village. In one of the rural districts (Phek), the customary tribal organization put a strict ban on bringing adult pigs into the district. The Phek district is remotely located but ASF was first reported from here indicating that ASF may be spreading mechanically or by smuggling of ASF-infected/survivor pigs or wild boar as reported earlier (21, 22). There is an urgent need to train the pig producers in scientific pig management including health. Also, the pig trade must be regulated to control the spread of ASF in the region.

In the study region, the majority of the respondents kept pigs for fattening purposes in *kutcha* pig sheds mostly located on the roadside. Fattener pig farms depend on external sources for the supply of piglets; hence, these farms are at increased risk of introducing the disease on the farm. Keeping pigs on the roadside exposed them to frequent visitors, poultry, dogs, and wild animals which are known carriers of ASF (6, 23). Maintaining clean and hygienic pig pens is difficult in wooden-made pig sheds. Also, spring, river, or rainwater may be contaminated with carcass, manure, urine, and other wastes which may further spread ASF and other contagious diseases (4, 22). Improvement in pig housing and its location along with regular cleaning with disinfectant should be promoted.

In the urban districts, farmers reared more crossbred pigs while in the rural districts, farmers kept more local pigs. The urban districts were the hot spot for live pigs and pork trade (5). Traders operating in the urban center (Dimapur) were bringing live pigs from different sources (approximately 1,000 pigs per day), mostly crossbred pigs, from North and South India (3,000 km away) and then bulking for sale to farmers or butchers or retailers of the entire state (field observation). In the previous study, Ma et al. (16) reported that pig density and transportation of live pigs and pork (24) products are important predictors of ASF outbreaks. This may be because of the fact that pigs will come into contact with sources of ASF infection as pig density and pig movement increase. Rural farmers preferred local pigs because local pigs are more adapted to their climate, more resistant to diseases, and require fewer inputs (20, 25). Hunting of wild boar was also a common practice in the study region, more so in rural areas. The potential role of wild boar in the spread of ASF in this region, considering its population and wide habitat, needs to be investigated. The respondents in the urban districts had heard of ASF, but they were unaware of the clinical signs and symptoms of ASF and disease transmission and its prevention. In the rural districts, respondents had not heard about ASF. It is important to mention here that ASF outbreaks had already been reported from Dimapur, Kohima, and Phek districts. During our study, respondents confirmed that there were large-scale deaths of pigs in their villages; however, this was not reported to the veterinary department. Blome et al. (6) reported that ASF outbreaks in Asia have revealed the weakness of the production system including poor veterinary services. Pig producers should be convinced to make isolated pig farms in the backyard of the house and water should be disinfected before use in the piggery. It is also recommended that pig density should be reduced in the urban district along with the regulation of pig movement. Pig farmers should be made aware of the danger of hunting for wild boar with respect to the spread of ASF along with the strengthening of veterinary extension services.

On-farm biosecurity was poorly implemented or not implemented by the respondents in both the urban and rural districts. Pig farms were not fenced and did not have footbaths at the entrance. There was no restriction on the movement of visitors to the pig farms and respondents did not quarantine the sick or newly purchased pigs. Gogin et al. (26) and Ma et al. (16) reported that human factors account for more than natural environmental factors for ASF occurrence. Although daily cleaning of pig pens was reported by the majority of the farmers, only a few respondents used disinfectant. Also, the safe disposal of dead pigs and manure was not followed by the majority of the respondents. Davies et al. (27) reported that the ASF virus remains infectious for almost 4 (urine) or 3 (feces) days at 37°C and thereby a potent source of the spread of ASF.

Pigs and piglets were purchased from unknown sources mostly from the weekly markets whose disease status is unknown. The sale or slaughtering of sick or dead pigs was common to avoid economic losses. In Dimapur districts, traders and butchers smuggled dead pigs in the night from adjoining areas and sold them in the market. Most of the respondents were engaged in pig trade (sold/purchased) in the past 12 months and there was frequent movement of live pigs. In previous studies, it was reported that areas with high volumes of pig movement and sourcing pigs from unknown sources during disease outbreaks further led to the spread of the disease (14, 19, 20). Also, the role of ASF-survivor pigs and the shedding of the virus for a long time has been reported (6); however, this aspect has been overlooked in India and needs scientific consideration.

The majority of the respondents reported that there was no waste during and after the slaughter of the pigs. However, in-depth questioning revealed that intestinal contents were discarded openly or in a drain. Traditionally, tribal people do the slaughtering in their homes and every part of the slaughtered pig is consumed including intestine, skin, and blood. However, after slaughter, the carcass is washed with water, and the same is discharged into the open area or stream. Blood is also offered to the live pigs in feed. It was also found that before cooking pork, it was washed thoroughly, and the wastewater is given to pigs along with feed. Swill feeding was a common practice in the study region. It was earlier reported that indirect transmission of ASF may occur when pigs consume swill containing infected material (28). Blome et al. (6) reported that the ASF virus is highly stable in the environment and raw pork, and careless use of porcine materials as a protein source for pigs will accelerate the spread of the epidemic. Ouma et al. (13) found that smallholder pig producers are reluctant to take up biosecurity measures if not incentivized economically to counter the increased cost. Besides, pig producers in developing countries are likely to prioritize income generation and food supply above disease prevention measures (29). Improving the on-farm as well as community biosecurity measures are important safeguards for smallholder pig farms against ASF in the absence of any licensed vaccine and effective treatment (6, 13, 14, 20, 23).

The strengths of this study were the large sample size, the selection procedure of households, and the local interviewers who knew the local dialect. We also ensured that interviewers were not to be related to the households surveyed. Although districts and blocks were purposively selected for the study, villages and households were randomly selected, thereby maintaining the random sampling framework. The large size of households in the study ensured that the study is representative of the population at large. Also, the study area is the core pig farming region of India and has a long porous international border, thus suitable for the spread of transboundary animal diseases.

The limitation of the study includes the response bias of the respondents. Response bias is difficult to eliminate from the study as some respondents might have given inaccurate answers knowingly to hide their behavior (7, 19). Also, the study uses household interviews by the research team, thus bringing in professional bias. However, this was minimized by engaging four interviewers, a design of simple and clear questions, and close-ended responses. In the study, we did not survey the pig farm having sick animals. This was done to avoid spreading the infection between the farms. This may have caused biases in the reported management and biosecurity practices. However, taking a large sample size, as was in the current study, negates such bias.

## 5. Conclusion

In summary, this study found out that the study region has a backyard pig production system with the use of outdated technologies that increase the risk of spread of infectious ASF and other infectious diseases. Pigs are confined in wooden sheds which are mostly located on the roadside. There is a significant volume of unregulated pig trade and pig movement in the region. In the study region, the presence of wild boar in the forest along with regular hunting by villagers presents another challenge to control ASF occurrence. On-farm biosecurity measures, disease diagnostic facilities, and veterinary extension services need to be strengthened. There is an urgent need to enhance the awareness of different stakeholders regarding the spread and control of ASF and other infectious diseases.

## Data availability statement

The original contributions presented in the study are included in the article/supplementary material, further inquiries can be directed to the corresponding author.

## Ethics statement

The animal study was reviewed and approved by Institute Animal Ethics Committee of ICAR Research Complex for NEH Region, Umiam, Meghalaya, India. Written informed consent was obtained from the owners for the participation of their animals in this study.

## Author contributions

Conceptualization: MS, MB, NP, RP, and RM. Methodology: MS, RY, and RK. Formal analysis and investigation: MS, NP, and PJ. Writing—original draft preparation: MS and RP. Writing—review and editing and funding acquisition: MS. Resources: HK and MB. Supervision: HK and VM. All authors contributed to the article and approved the submitted version.
